# Engineering Compounds for the Recovery of Critical Elements from Slags: Melt Characteristics of Li_5_AlO_4_, LiAlO_2_, and LiAl_5_O_8_

**DOI:** 10.1021/acsomega.4c00723

**Published:** 2024-05-28

**Authors:** Sven Hampel, Iyad Alabd Alhafez, Thomas Schirmer, Nina Merkert, Sophie Wunderlich, Alena Schnickmann, Haojie Li, Michael Fischlschweiger, Ursula Elisabeth Adriane Fittschen

**Affiliations:** † Institute of Inorganic and Analytical Chemistry, Clausthal University of Technology, Arnold-Sommerfeld-Straße 4, Clausthal-Zellerfeld 38678, Germany; ‡ Institute of Applied Mechanics, Clausthal University of Technology, Arnold-Sommerfeld-Straße 6, Clausthal-Zellerfeld 38678, Germany; § Department of Mineralogy Geochemistry Salt Deposits, Clausthal University of Technology, Adolph-Roemer-Straße 2A, Clausthal-Zellerfeld 38678 Germany; ∥ Technical Thermodynamics and Energy Efficient Material Treatment, Clausthal University of Technology, Agricolastraße 4, Clausthal-Zellerfeld 38678, Germany

## Abstract

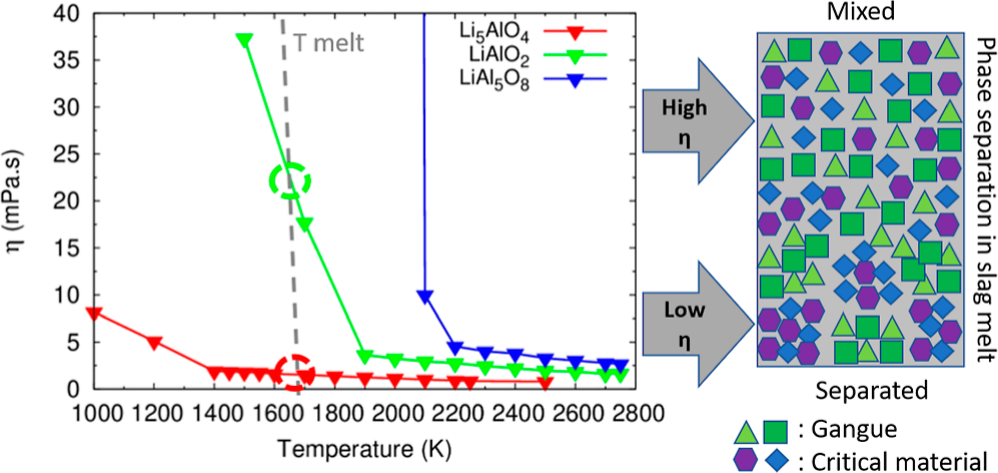

Engineered artificial minerals (EnAMs) are the core of a new concept of designing scavenger compounds for the recovery of critical elements from slags. It requires a fundamental understanding of solidification from complex oxide melts. Ion diffusivity and viscosity play vital roles in this process. In the melt, phase separations and ion transport give rise to gradients/increments in composition and, with it, to ion diffusivity, temperature, and viscosity. Due to this complexity, solidification phenomena are yet not well understood. If the melt is understood as increments of simple composition on a microscopic level, then the properties of these are more easily accessible from models and experiments. Here, we obtain these data for three stoichiometric lithium aluminum oxides. LiAlO_2_ is a promising EnAM for the recovery of lithium from lithium-ion battery pyrometallurgical processing. It is obtained through the addition of aluminum to the recycling slag melt. The high temperature properties spanning from below to above the liquidus temperature of three stoichiometric Li–Al–Oxides: Li_5_AlO_4_, LiAlO_2_, and LiAl_5_O_8_ are determined using molecular dynamic simulations. The compounds are also synthesized via the sol–gel route. The Li^+^ ion exhibits the largest diffusivity. They are quite mobile already below the liquidus temperature, i.e., for LiAlO_2_ at *T* = 1700 K, the diffusion coefficient of the lithium ion equals *D* = 3.0 × 10^–9^ m^2^ s^–1^. The other ions Al^3+^ and O^2–^ do not move considerably at that temperature. The diffusivity of Li^+^ is largest in the lithium-rich compound Li_5_AlO_4_ with *D* = 32 × 10^–9^ m^2^ s^–1^ at 2500 K. The lower the viscosity, the higher the lithium content. The Li_5_AlO_4_ exhibits a viscosity of η = 2.2 mPa s at 1328 K which matches well with the experimentally determined 2.5 mPa s at this temperature. The viscosity of LiAlO_2_ at 1800 K is more than two times higher. These data sets can help to describe the melts on a microscopic level and understand how the melt properties will change due to gradients in the Li/Al concentration.

## Introduction

### Recovery of Critical Materials from Slags

**Figure 1:**
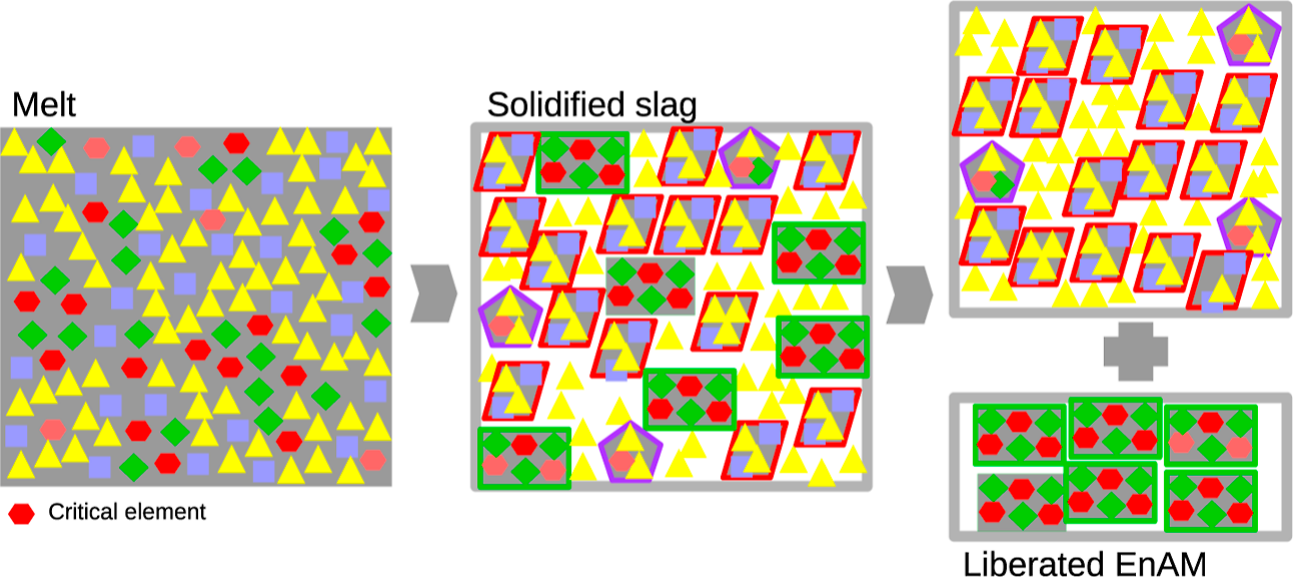
Concept of EnAM. Starting from the left: initially the melt is represented by a high degree of dispersion of the constituting cations and the oxygen counterions. Tetrahedral cation-oxygen polytops like [SiO_4_]^*n*−^ can form networks by oxygen bridging, here represented in yellow triangles. The purple squares represent cations which do not form networks, e.g., Ca^2+^. The critical cation is presented as red hexagons, e.g., Li^+^. To drive the critical element in a solitary phase, a mineral forming additive is added, here green diamonds, e.g., Al^3+^. After solidification, the EnAM ideally binds all critical elements (middle image). Here, the rectangles constituted of red and green elements. These are separated in the last step from the “gangue” material.

The recovery of critical materials from waste streams has become a major concern in industrialized regions of the world with limited primary resources. The European Union has defined a list of materials critical for its economy and accessed their supply risk for the first time in 2011. It has been updating this list ever since.^1^ From the correlation of both, specific elements and materials were considered critical and of high priority for recycling efforts and prospecting. For example, heavy rare earth elements are considered very critical.^2^ With respect to increasing use of electric vehicles, cobalt and, recently, lithium have been rated critical materials.^3,4^ Various pyrometallurgical routes have already been developed, e.g., by the company Umicore (bath T of approx. 1723 K).^5^ As these routes only concentrate on the recovery of the more noble elements such as cobalt, nickel, and copper, it is accepted that elements with high oxygen affinity (e.g., lithium, rare earth elements, tantalum) migrate into the slag.^6,7^ The maximum yield of lithium in the slag is reported to be >80 wt % of the total Li of the recycled batteries.^8^ However, a substantial amount is lost through evaporation. Although recycling processes including hydrometallurgical leaching of Li from the battery recycling slags have been developed,^9^ commonly, the slags are reused in construction work and the critical elements incorporated into them are lost for recovery. Leaching procedures are sometimes discussed.^10^ A new highly promising concept to facilitate a cost- and resource-efficient recovery is to bind these elements in minerals that can be easily separated and enriched from the slag. This new approach, which is currently being investigated in various research projects, is known as the “engineered artificial minerals” method. The term most often used to refer to these minerals is “engineered artificial minerals” (EnAM).^2,6,11^ The concept of EnAM formation and liberation is illustrated in Figure 1.

An efficient liberation and separation in general require the EnAM to be the only/main compound formed from the melt bearing the critical element. Meaning, the ratio of the fraction of the element in the slag matrix (ElSM) to the fraction in the EnAm compound (ElEnAm) is minimized: ElSM/ElEnAm = min (optimum: 0). In the optimum case, the crystal morphology is isometric and the crystallite size is maximized, as small dendritic or needle-shaped crystals make liberation more difficult.^6,12^ Though there is a maximum in flotation, usually 20–40 μm,^13^ comminution will allow to liberate particle of this size, when the grain size is larger. If possible, an EnAm compound should be an early or first crystallizate, as this ensures that the crystals have sufficient space to grow and the target element cannot be incorporated into any other compound. Accordingly, it is necessary to synthesize compounds from the melt that crystallize first at high temperatures and exhibit no competing phases.

### Solidification of Slags

The solidification from these complex melts is not well understood. Though the thermodynamic prediction of phase transitions is fundamental to understand compound formation, solidification from high temperature melts <1273 K is additionally governed kinetically.^14^ The viscosity of the melt and the individual ion diffusivity play a vital role in this process.^15,16^ In viscous melts, solidification may occur without predicted crystal formation. High viscosity and low ion diffusivity can limit the support of the constituent elements during crystal growth. The processes in the melts can be quite complex. During cooling, phase separation in the melt can occur in the form of liquid–liquid separations as well as solid–liquid separation.^14,17,18^ The phase separation gives rise to composition gradients in solid–liquid separation and sharp compositional interfaces in liquid–liquid separation. Different composition translates into different viscosity, and melt-chemistry, e.g., redox-potential.^19,20^ Compounds like Fe_2_O_3_ reduce the viscosity significantly due to depolymerization of the silicate network and increase the oxygen diffusivity by an order of magnitude.^21,22^ As a first approximation, we propose to understand the melt as a regime of phase transitions with microscopic areas of simple composition. The description of those, especially its viscosity and ion diffusivity, will facilitate deeper understanding of the solidification.

### LiAlO_2_ a Potential EnAM for Lithium Recovery

**Figure 2:**
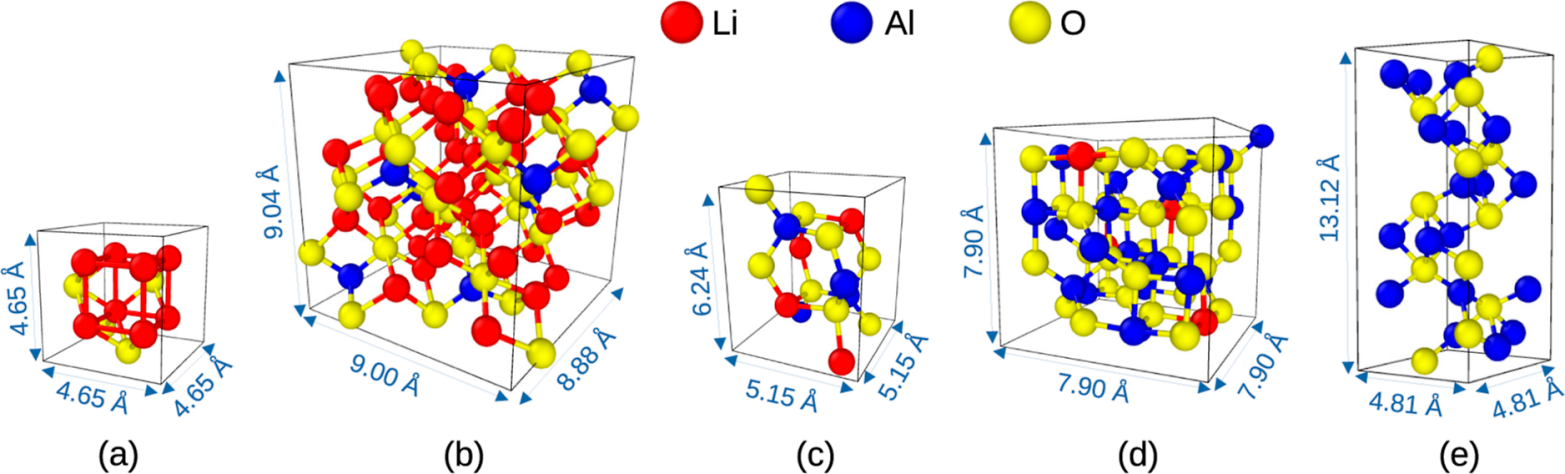
Crystal structure of the unit cell of (a) Li_2_O, (b) Li_5_AlO_4_, (c) LiAlO_2_, (d) LiAl_5_O_8_, and (e) Al_2_O_3_. Information about the crystal system and space group is given in Table 2.

The lithium aluminum oxide LiAlO_2_ is an early crystallizate. It has been observed to form from silicate recycling melts when aluminum is added. It bound nearly all lithium and exhibited excellent characteristics for the subsequent crushing and sorting and hence was suggested as a potential EnAM for lithium recovery.^6^ Flotation studies using novel collectors showed good performance of up to 89% recovery of lithium aluminate separating it from spodumene.^23^ High diffusivity of oxygen potentially increases the oxidation state of redox-active elements like manganese, which has a major influence on the formation of the EnAM LiAlO_2_.^6,11^ Accordingly, knowledge about the melt, e.g., diffusivity and viscosity, has the potential to predict and design compound formation in metal oxide melts. Molecular dynamics (MD) simulations are potent tools to study the high-temperature metal oxide properties. MD studies found that networks present in TiO_2_–FeO–Na_2_O melt significantly impact viscosity and solidification processes in slags.^24^ MD simulations of lithium aluminum oxides have been used to study the defect chemistry and sophisticated potentials could be developed due to the simple crystal structures and strong ionicity among ternary lithium-containing oxides.^25,26^ However, less attention was paid to transport properties, such as viscosity. Here, we study individual ion diffusivity and viscosity of Li–Al–O-compounds with different Li/Al ratios according to their thermodynamically predicted phase diagram. These are important properties that help to understand microscopic conditions during solidification. The selected compounds differ in the Li/Al ratio, and from lithium-rich to poor, the compound stoichiometry is Li_5_AlO_4_, LiAlO_2_, and LiAl_5_O_8_. The unit cells of these compounds and the pure endmembers of Li_2_O and Al_2_O_3_ are shown in Figure 2. The diffusivity of Li^+^, Al^3+^, and O^2–^ and viscosity at temperatures between 1000 and 1900 K were determined using MD simulations.

## Experimental and Computational Methods

### Molecular Dynamic Simulations

All the MD simulations are performed with the open-source LAMMPS code.^27^ Periodic boundary conditions are employed, and the time step in all of the simulations is 1 fs. In the system Li_2_O–Al_2_O_3_, three stable lithium aluminate compounds are studied: Li_5_AlO_4_, LiAlO_2_, and LiAl_5_O_8_ beside Li_2_O and Al_2_O_3_. In the present study, charged particles interact via the long-range Coulomb potential supplemented by the short-range Buckingham potential (eq 1)
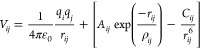
1here *i* and *j* denote an interacting pair of two ions among lithium, aluminum, and oxygen separated by a distance *r*_*ij*_, ε_0_ stands for the dielectric constant. The Buckingham potential parameters are taken from Kuganathan et al.^25^ and are listed in Table 1. Oxygen atoms’ polarizability is included using the core–shell model,^28^ in which a harmonic spring connects the positive core (charge +0.8) with the negative shell (charge −2.8). The adiabatic core–shell model by Mitchell and Fincham^29^ is used with the CORESHELL package within LAMMPS.

**Table 1:** Interatomic Potential Parameters Used in the Atomistic Simulations of Li–Al–O Systems Using a Core–Shell Model with Y Electrons and Spring Constant K

interaction	*A* (eV)	ρ (Å)	*C* (eV Å^6^)	*Y* (e)	*K* (eV Å^–2^)
Li^+^–O^2–^	632.1018	0.2906	0.00	1.000	99999
Al^3+^–O^2–^	1109.92381	0.31540	0.00	3.00	99999
O^2–^–O^2–^	12420.5	0.2215	29.07	–2.80	31.0

The crystal structures of the five compounds at the initial state are summarized in Table 2 and shown in Figure 2. Size effects of the simulated systems are expected to be small using properly large cells and by applying periodic boundary conditions in all three dimensions. At first, the samples are relaxed at a temperature of around 300 K using an isothermal–isobaric (*NPT*) ensemble for 20 ps. After relaxation of the samples, the compounds are heated up to the target temperature which is dominantly 2500 K. This high temperature was chosen to ensure that all compounds are in liquid state. At the target temperature, the samples are relaxed again for 500 ps. All visualizations as well as the analysis of the radial distribution function (RDF) and the coordination analysis are done by the software tool OVITO-PRO.^30^

**Table 2:** Compounds Present in the Li_2_O–Al_2_O_3_ System Used in the Simulations, Their Crystal Systems, Space Groups, Dimensions, and the Number of Atoms^a^

compound	crystal system	space group symbol	dimensions (Å)	number of atoms
Li_2_O	cubic	*Fm*3̅*m*	56 × 56 × 56	20,736
Li_5_AlO_4_	orthorhombic	*Pbca*	62 × 63 × 63	27,440
LiAlO_2_	tetragonal	*P*4_1_2_1_2	57 × 57 × 69	21,296
LiAl_5_O_8_	cubic	*P*4_3_32	71 × 71 × 71	40,824
Al_2_O_3_	trigonal	*R*3̅*c*	64 × 66 × 65	32,380

a Initial atomic coordinates were taken from ref (31). The corresponding crystal structures are found in ref (32–36).

### Calphad Model

Calculation of phase diagram (CALPHAD) is a method that integrates experimental information, e.g., thermal and caloric material properties, phase diagram data, as well as data from atomistic modeling to establish thermodynamic models and parameters such that the Gibbs energies for respective phases for various materials systems can be predicted. Applications are for instance in the understanding of material’s microstructure and solidification predictions.^37^ Particularly, in terms of lithium-containing oxide material systems, CALPHAD is widely applied for calculating and predicting the phase diagrams and serves as input for nonequilibrium solidification modeling.^8,12,38−42^ In this work, thermodynamic models and thermodynamic database of the Li_2_O–Al_2_O_3_ system are applied according to Konar et al.^42^ Based on this, essential phase information is provided for synthesizing different lithium-containing samples.

### Chemicals

Lithium hydroxide, aluminum nitrate nona hydrate, citric acid, 65% nitric acid, 25% ammonia, 95–97% sulfuric acid, and 35% hydrogen peroxide were obtained in pro analysis quality from Carl Roth. Dilutions were performed with ultrapure water (>18.2 MΩ cm, Purelab Flex 4, ELGA Veolia).

Glazed porcelain crucibles (15 mL, wide form) were purchased from IDL for the sintering of the powders. Crucibles were cleaned in boiling 10% nitric acid for 4 h and were heated to 1273 K for 2 h before usage.

Argon (99.996% purity, Linde) was used for sintering in an inert atmosphere.

### Instrumentation

Powder X-ray diffraction analysis was performed with a PANalytical X’PERT PRO diffractometer with Co Kα excitation (40 kV, 40 mA) as the scatter sample in Bragg–Brentano geometry. Identification of the phases was evaluated with the PDF-2 ICDD database.^43^ Rietveld refinement was performed with the program package Fullprof.^44^

Sintering of the powders prepared by sol–gel synthesis was performed in a Nabertherm L 3/11 furnace with minimal air supply. Preparation of Li_5_AlO_4_ took place in a Naberthem LHT 02/18 furnace with automatic gas supply set to 200 L h^–1^ argon at 1.2 bar. Heating ramps were set for both furnaces to 10 K min^–1^, while cooling was limited by the furnace specifications. Microwave-assisted digestions were performed in 10 mL quartz tubes in a CEM discover SP-D 80 microwave (300 W). Digestions were carried out in triplicate with about 25 mg of substance each using a mixture of 4 mL of sulfuric acid and 0.5 mL of hydrogen peroxide. The ramping temperature was set to 473 K with a 10 min ramping time and 20 min holding time. The digestion was repeated with an additional 0.5 mL of hydrogen peroxide added. The resulting solutions were diluted to a final volume of 25 mL for inductively coupled plasma optical emission spectrometry (ICP-OES). Standard additions for aluminum and lithium, respectively, were done in order to reduce matrix effects during the determination.

ICP-OES was performed with a Varian Vista-MPX instrument equipped with a vertical quartz torch (radial plasma) with a V-gap atomizer in a PTFE chamber. Torch power was set to 1.2 kW with 15 L min^–1^ argon for the plasma, 1.5 L min^–1^ argon around the torch, as well as 0.88 L min^–1^ for the atomizer. Observation height was set to 12 mm with evaluation of lithium 670.78 nm and aluminum 396.15 nm lines.

Determination of the viscosity of Li_5_AlO_4_ was obtained by using a Hesse Instruments EM301 heating microscope. A 3 mm diameter cylinder of the compound with a height of 3 mm was heated to 673 K at an 80 K min^–1^ heating rate followed by 7 K min^–1^ toward the maximum temperature of 1773 K.^45^ The half sphere temperature was determined to be 1328 K. In another run, another cylinder of sample material was heated using the same rate as described before but with an end temperature of 1328 K, which is held for 60 min. The heights and baseline of the shadow profile were used to calculate the squared half height width of half of the profile. This value is plotted over the elapsed time, the obtained slope of 3097.1 μm^2^ s^–1^ of the linear regression *R*^2^ = 0.9439 was interpreted as diffusion coefficient *D*. The viscosity was then obtained using eq 2.
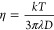
2

In eq 2, η is the viscosity, *k* the Boltzmann constant, *T* the absolute temperature, *D* the diffusion coefficient, and λ is the translation distance of the diffusing ion which is always considered to be 2.8 Å, the diameter of oxygen.^46−48^

### Compound Preparation

Synthesis of LiAlO_2_ and LiAl_5_O_8_ was performed with a modified Pechini method similar to Blank et al.^49^ Lithium hydroxide and aluminum nitrate nona hydrate in corresponding mole fractions (total amount of substance = 30 mmol) were dissolved in about 30 mL of 20% nitric acid. Citric acid in 10 mol % excess was added, and the mixture was set to a neutral pH at reflux temperature with 25% ammonia solution. Excess water was evaporated upon the self-ignition of the mixture. The resulting powders were sintered at 1273 K for 2 h in air.

Due to the increased evaporation of lithium with smaller particle sizes, the sol–gel synthesis was not suitable for Li_5_AlO_4_ as only LiAlO_2_ resulted. A bulk powder approach was used instead, with mixing 3.75 g (10 mmol) aluminum nitrate nona hydrate with 1.31 g (55 mmol, 10 mol % excess) lithium hydroxide. The mixture was sintered at 1023 K for 24 h in argon atmosphere.

## Results and Discussion

### Lithium Aluminum Oxides

With phase equilibrium thermodynamics, the eutectic composition of the Li_2_O–Al_2_O_3_ system is determined. By applying CALPHAD^37^ with a database described in previous studies,^38,40,42^ the eutectic composition is identified to be 0.22. Additionally, the CALPHAD modeling predicts the compounds LiAlO_2_ and Li_5_AlO_4_ which are formed during solidification at this certain composition. Based on this information, the synthesis procedure was set up in this work, summarized in Table 3. The compositions of Al/Li of 0.17, 0.50, and 0.83 yield the stoichiometric compounds Li_5_AlO_4_, LiAlO_2_, and LiAl_5_O_8_ that are selected for this study. The predicted high temperature aluminate LiAl_11_O_17_ which is found at high aluminum concentrations has no common stability zone with the EnAM LiAlO_2_. Additionally, an extensive study on the Li_2_–Al_2_O–3 system by De Abreu et al. found no evidence for the phase formation and decomposition of LiAl_11_O_17_ by differential thermal analysis (DTA) as well as no microstructural evidence of an eutectoid decomposition of it using Scanning Electron Microscopy.^50^ The compound was, therefore, not considered in the models at this point. The compounds were synthesized via the sol–gel route, as described in the experimental section. The targeted composition was evaluated by ICP-OES. The elemental balance of target and final compound are displayed in Table 3.

**Table 3:** Fraction of Lithium Oxide and Aluminum Oxide in the Stoichiometric Compounds; Theoretical and Synthesis

compound	target Li_2_O/Al_2_O_3_	product Li_2_O/Al_2_O_3_
Li_5_AlO_4_	0.833:0.167	0.829:0.171
LiAlO_2_	0.500:0.500	0.496:0.504
LiAl_5_O_8_	0.167:0.833	0.158:0.842

**Figure 3:**
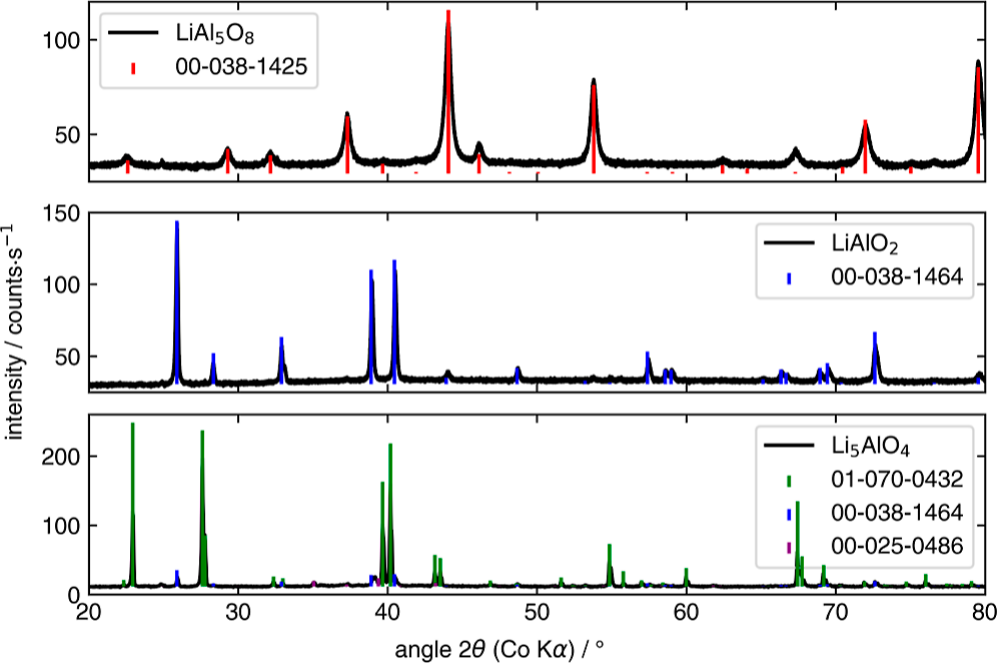
Diffractograms of LiAl_5_O_8_, LiAlO_2_, and Li_5_AlO_4_ with references from PDF2 database.^43^ The corresponding crystal structures are given in refs (34−36 and 51).

The elemental determination confirms the expected stoichiometries for all three compounds. The structure of the compounds was validated by powder X-ray diffraction. The diffractograms exhibit a low background and, in general, only the expected reflexes. The stoichiometry as well as the diffractogram confirm the synthesis of LiAl_5_O_8_, LiAlO_2_, and Li_5_AlO_4_ (Figure 3). Rietveld refinement of the obtained Li_5_AlO_4_ reveals a composition of 85.2% Li_5_AlO_4_, 6.8% LiAlO_2_, and 8.0% LiOH· H_2_O (see Figure S1). Further reflexes with minor intensities could not be assigned. The viscosity of Li_5_AlO_4_ was studied for comparison with the MD results.

Viscosity and ion diffusivity are decisive information for gaining a better understanding of the solidification of melts. The diffusivity and viscosity were calculated from MD simulations of Li_5_AlO_4_, LiAlO_2_, and LiAl_5_O_8_ at temperature spanning from solid state to the phase transition and the molten state. Experimentally, the viscosity and melting point of the relatively low melting compound Li_5_AlO_4_ were accessible using melt-microscopy.^45^ The individual ion diffusivity was exclusively available from the MD simulations. In the following section, we will describe the results obtained from MD simulations, i.e., diffusivity of the ions and viscosity of the compounds at high temperatures.

### Individual Ion Diffusivity

**Figure 4:**
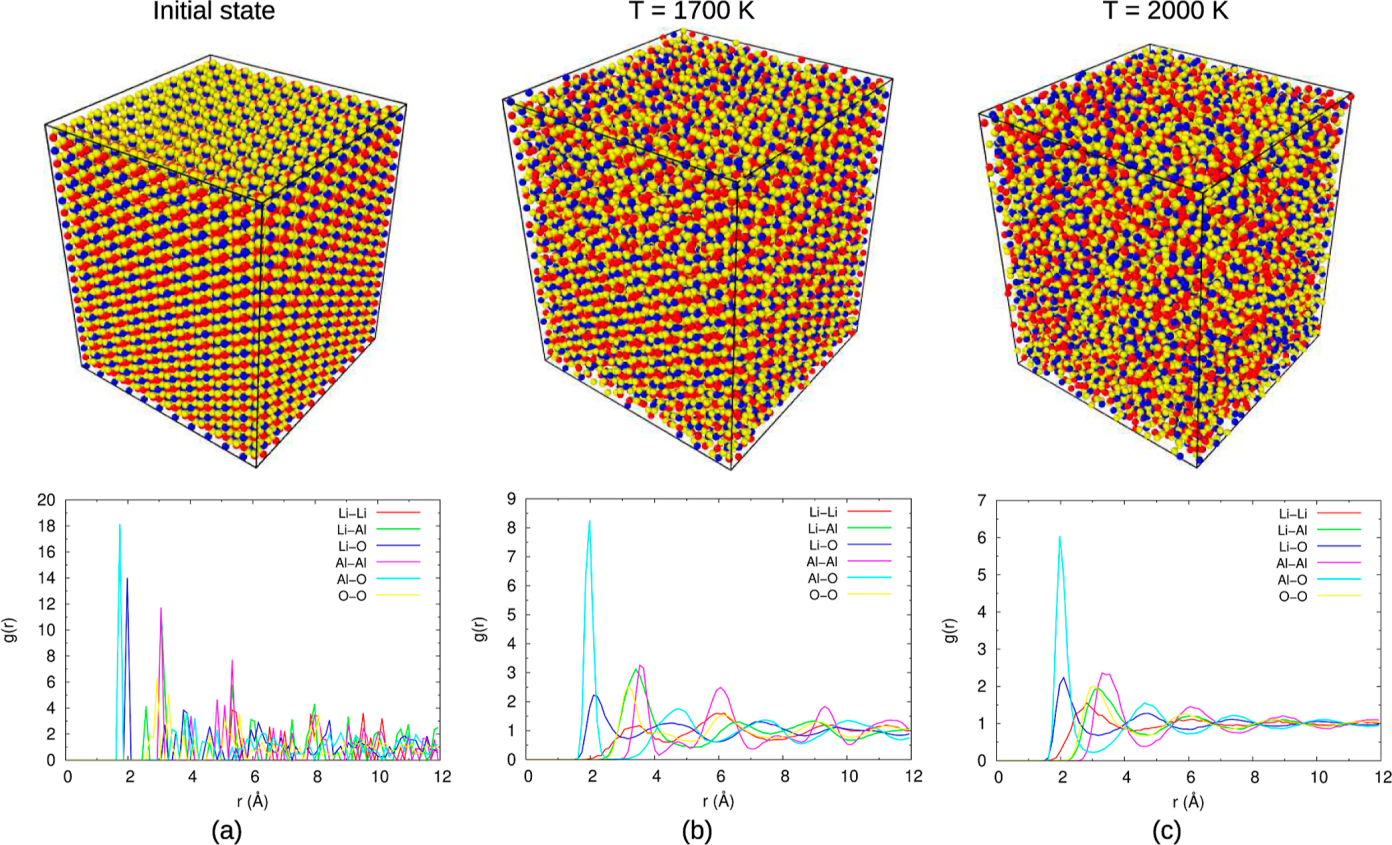
Structural snapshots and RDF of LiAlO_2_ at (a) the initial state at *T* = 300 K, (b) *T* = 1700 K, and (c) *T* = 2000 K.

The MD simulations allow studying the position of the individual ions Li^+^, Al^3+^, and O^2–^ over time. The simulations were performed for all three compounds, and at the end of this subsection, ion diffusivity in all three compounds are compared. However, for a concise presentation, we mainly illustrate the results for LiAlO_2_, which has been already identified as potential EnAM. The corresponding illustration for the other compounds are shown in the Supporting Information (Figures S2–S8). The higher the temperature, the more random the positions of the ions are. This can be illustrated by snapshots of the simulated cube and the RDF. In Figure 4, this is illustrated for LiAlO_2_ at 300 K, 1700 K which is close to the liquidus temperature and 2000 K which is above the liquidus temperature. The snapshots visualize the degree of disorder increasing with temperature. The RDF at 300 K exhibits the interionic distances as expected in a LiAlO_2_ crystal (space group *P*4_1_2_1_2). The distances of the individual ion pairs are described by quite narrow distributions (see colored lines in Figure 4). At 1700 K, the distributions have significantly broadened, and at 2000 K, the RDF displays the characteristics of a liquid.

It is visible in the RDF that some distance distributions broaden more than others with an increase in temperature. For example, the Al–O distance distribution remains relatively narrow, whereas distances between Li^+^ and other ions become more random. This is also seen in the RDFs of the other two compounds (Figures S3–S6). The mobility of the ions is described by their mean square displacement (MSD). It can be computed for each individual ion and then averaged over all ions of the species, and it is a measure of their diffusivity. The diffusion coefficient *D* is calculated from the mean-square displacement of the lithium, aluminum, and oxygen ions according to eq 3.
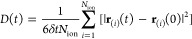
3

In the equation, *t* denotes the time and the above sum is carried out over the number of ions *N*_ion_.

**Figure 5:**
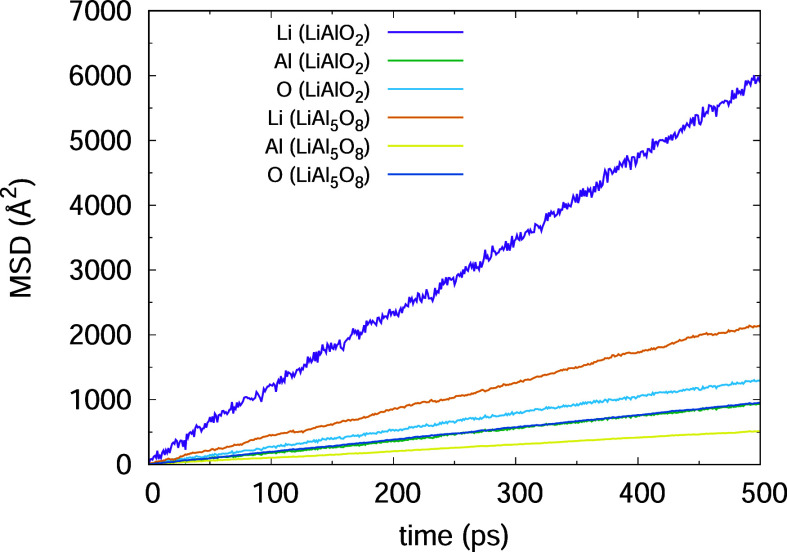
Mean-square displacement of Li^+^, Al^3+^, and O^2–^ in LiAlO_2_ and LiAl_5_O_8_ at *T* = 2500 K.

**Figure 6:**
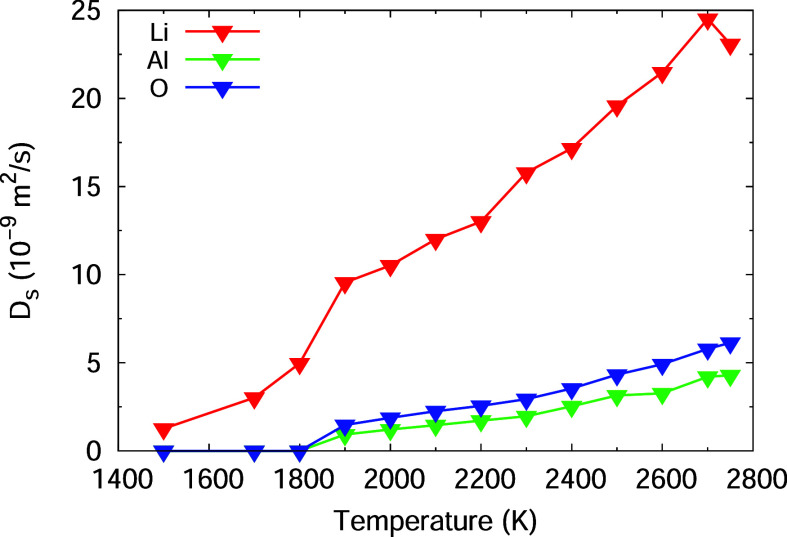
Diffusion coefficient of Li^+^, Al^3+^, and O^2–^ in LiAlO_2_ at temperatures from 1500 to 2700 K.

The MSD of all three lithium aluminum oxides at 2500 K clearly shows that Li^+^ is the most mobile of the ions, which is shown here for LiAlO_2_ and LiAl_5_O_8_ (Figure 5, for each component see S2, S4 and S7). This is well in agreement with the literature, where the lithium-ion activation energy was found being lower compared to other ions, i.e., 0.53 eV.^25^ Our approach allows us to determine diffusivity of all three ions during phase transition of the Li–Al–Oxides, shown here for LiAlO_2_ (Figure 6, for the others see S5–S8) where a rapid increase of all ions’ diffusion between 1800 and 1900 K is observed. Some results of LiAlO_2_ are shown in Table 4.

**Table 4:** Diffusion Coefficients *D* (10^–9^ m^2^ s^–1^) of the Constituting Ions of LiAlO_2_ at Different Temperatures at the Phase Transition and Lithium Diffusion Coefficients from the Literature^52^

ions in LiAlO_2_	*T* = 1700 K	*T* = 1800 K	*T* = 1900 K	*T* = 2000 K
Li	3.0	5.0	9.6	10.5
Li (Jacobs et al.)^52^	8.9	10.9	13.0	15.2
Al	0	0	0.9	1.2
O	0	0	1.5	1.9

**Figure 7:**
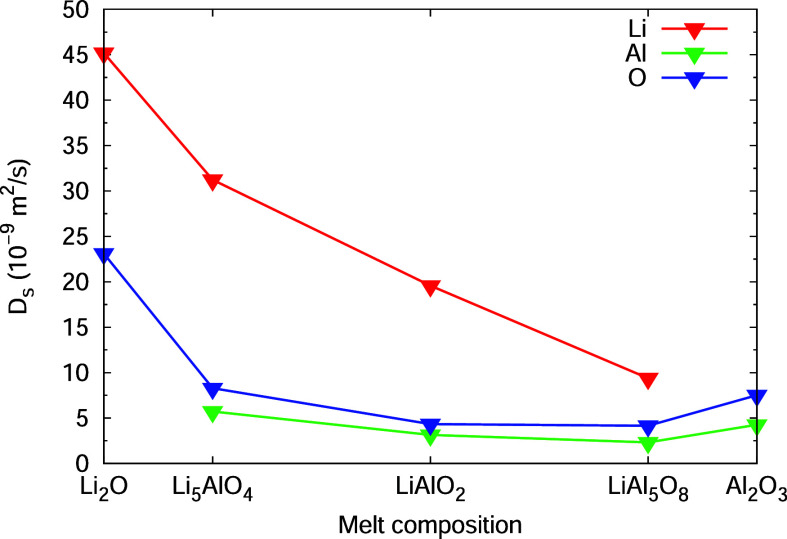
Composition dependence of the self-diffusion coefficient of Li^+^, Al^3+^, and O^2–^ at *T* = 2500 K.

These results match quite well with the simulations of Jacobs et al.^52^ They studied lithium diffusion in LiAlO_2_ with respect to its application in nuclear fusion. Here, however, we examine besides LiAlO_2_ the higher and lower lithiated compounds Li_5_AlO_4_ and LiAl_5_O_8_ which according to the thermodynamic model can form in lithium-ion battery recycling slags depending on the actual composition. From the MD simulations of all compounds, it is evident that the lithium ions are more mobile in the lithium-rich compounds Li_5_AlO_4_ and LiAlO_2_ (Figure 7). Its diffusion coefficient at *T* = 2500 K in the lithium-poor LiAl_5_O_8_ is only approx. one-third (*D* = 12 × 10^–9^ m^2^ s^–1^) of the one in the lithium-rich Li_5_AlO_4_ (*D* = 32 × 10^–9^ m^2^ s^–1^). The mobility of aluminum ions and oxygen ions seams to decrease with decreasing lithium content; however, this is not significant.

Losses of lithium to the gas phase have been observed during the optimization of their Li–Al–oxide compound synthesis. An enrichment of approx. 70% of lithium in the flue dust is described in the literature in a small-scale lithium-ion battery pyrometallurgical recycling approach.^9^ Its relatively high diffusivity may contribute to its tendency to transfer from the molten phase to the gas phase.

### Viscosity Depending on Li/Al Ratio

**Figure 8:**
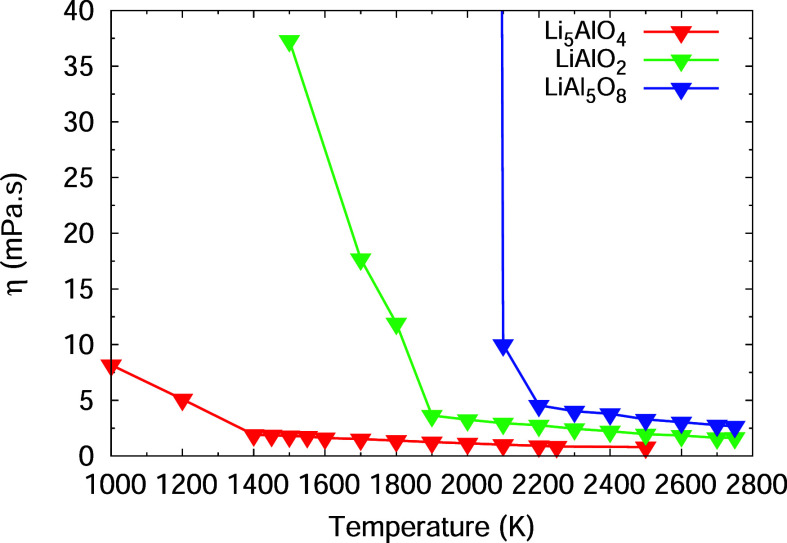
Viscosity of Li_5_AlO_4_, LiAlO_2_, and LiAl_5_O_8_ as a function of temperature.

The viscosity of the Li–Al-oxide discussed here is accessible from the diffusion coefficients obtained from the MD simulations (eq 3) using eq 2. The temperature dependence of the viscosity of the three Li–Al-oxides is shown in Figure 8 and Table 5. A strong decrease of the viscosity is observed above the liquidus temperature, which is lowest for Li_5_AlO_4_ and highest for the LiAl_5_O_8_ composition. It appears that the higher the lithium content of the melt, the lower the viscosity, which indicates that a high content of lithium promotes phase separations in the liquidus and with that can govern the formation of the solid compounds. Experimentally, the viscosity is available with high confidence for low melting Li_5_AlO_4_. It was determined to be 2.5 mPa s at 1328 K matching well the simulated viscosity at this temperature of 2.2 mPa s. The two other compounds did not show melting up to 1773 K accessible with the microscope. The shadow image of the pressed substances remained a constant size and shape at a hold temperature of 1773 K, which was held constant over half an hour. The observable high temperature behavior of the compound is in accordance with the CALPHAD and MD model. In addition to our CALPHAD prediction, this is further supported by CALPHAD-based phase equilibrium calculations conducted in ref (42). Furthermore, ref (53) confirms that the melting temperatures of the complex oxides LiAlO_2_ and LiAl_5_O_8_ are 2058 and 2188 K, respectively. De Abreu et al. determined peritectic melting of Li_5_AlO_4_ at 2222 K using the DTA.^50^

**Table 5:** Viscosity η (mPa s) Obtained from the Simulations for the Different LiAlOs

compound	*T* = 1700 K	*T* = 1800 K	*T* = 1900 K	*T* = 2000 K
Li_5_AlO_4_	1.5	1.4	1.25	1.1
LiAlO_2_	18	12	3.6	3.3
LiAl_5_O_8_	4980	3080	1900	880

The knowledge of the melt viscosity is highly valuable, as it influences the kinetics of solidification and can contribute to phase separations. Liquidus separation into immiscible phases can be predicted based on thermodynamics, taking the enthalpy and the entropy of mixing into account. If the enthalpy of mixing is positive, a critical temperature exists below which phase separation is spontaneous. Kinetic parameters, especially the viscosity, are decisive for phase separation actually to occur. Low viscosity will quickly yield a phase separation, whereas viscous melts will separate very slowly. Phase separation in molten slags has not been described in detail on a molecular level in the literature. Liquid–liquid immiscibility, is more extensively studied as a common phenomenon in glass forming melts.^18^ Actually, far more binary glass forming melts exhibit liquid–liquid immiscibility than exhibit homogeneous liquid behavior.^54^ The shape of the immiscibility volume in the system BaO–B_2_O_3_–SiO_2_ was determined from experimental data.^17^ Though, thermodynamics predict phase separation for many glasses, due to high viscosity, those are not always observed.^15,16^ High viscosity hampered studying the immiscibility in the B_2_O_3_–SiO_2_ system.^55^ Once separated, the phases exhibit different compositions, melting points, ion mobility, and viscosity. Liquid–solid phase separations in a Al–Si melt were also influenced by viscosity in a way, that the adhesion of the formed Si-crystals was supported by high viscosity.^56^ With respect to tailor EnAMs in slag systems, knowledge of ion mobility and viscosity of the potential compounds will be a key parameter, and it can be obtained by MD simulations as shown here (e.g., Figures 7 and 8).

### Individual Ion Coordination Number Depending on Composition

The experimental results agree with the findings of the simulations, which increases the confidence in the other simulated parameters at high temperatures. These are difficult to assess experimentally. The simulations can potentially yield further information on the melt structure. The mean coordination numbers are determined by extracting the positions *r* corresponding to the initial peaks in their respective RDF (Figure 4). One interesting observation is the increase in the mean coordination number of Li^+^ and Al^3+^ in the melt, when comparing the three compounds from high to low lithium contents. The mean coordination number of Li^+^ in Li_5_AlO_4_ is approx. 3 and increases to 4 in the LiAl_5_O_8_. The mean coordination number of Al^3+^ in Li_5_AlO_4_ is approx. 4 and increases to 5 in LiAl_5_O_8_. The coordination number in the melt can steer the structure of the solidified compound. Of course, the simulations have limitations; e.g., they do not account for elemental transitions to the gas phase. During the simulations, the number of elements is always conserved.

## Conclusions

In this work, we study melt characteristics of the three stoichiometric compounds in the Li_2_O–Al_2_O_3_ system, i.e., Li_5_AlO_4_, LiAlO_2_, and LiAl_5_O_8_. Our rational is that when we learn about viscosity of certain compositions and diffusivity of the constituent ions, conclusion helping the prediction of phase separation and solidification can be derived. We have chosen the Li–Al–O system because LiAlO_2_ has been identified previously as a promising phase to recover lithium from lithium-ion battery recycling slags. Initially, the eutectic composition in terms of LiAlO_2_ and Li_5_AlO_4_ is calculated under the CALPHAD framework. We have then simulated the MD of the three stoichiometric compounds at temperatures from below to well above the liquidus temperature and determined the viscosity of each compound with increasing temperature. Additionally, we determined the diffusivity of Li^+^, Al^3+^, and O^2–^ for each compound and at each temperature. The viscosity was well in agreement with those obtained from literature and from reference compounds synthesized by the sol–gel route. It can be concluded that Li^+^ already diffuses at temperatures below the liquidus. Its diffusion coefficient is about ten times higher than that of Al^3+^ at the melting point in, i.e., LiAlO_2_. This implies that the availability of Li^+^ is not a limiting factor in the crystal formation of Li–Al–O rich melts. The results of the MD simulations and the experiments show that the viscosity increases with a decreasing lithium content. This finding suggests that lithium rich melts are more fluid, and high lithium concentrations will promote liquidus phase separation, and it could be an interesting topic for thermodynamics in the future. In conclusion, we have gained valuable insight into the melt characteristics of the Li–Al-oxides, which will be important for understanding compound formation in slags with respect to recycling critical elements.

## Supporting Information Available

Rietveld refinement of LiAl_5_O_8_, and modeling results for the compounds LiAlO_2_, Li_5_AlO_4_, and LiAl_5_O_8_ (PDF)
