# Supplementary material for: Engineering Compounds for the Recovery of Critical Elements from Slags: Melt Characteristics of Li5AlO4, LiAlO2, and LiAl5O8
Source: ACS Omega. 2024 May 28;9(23):24584–92. doi: 10.1021/acsomega.4c00723 (PMC11170697; doi:10.1021/acsomega.4c00723)
Supplement: Supplementary file 1 — ao4c00723_si_001.pdf [file ao4c00723_si_001.pdf]

Supplementary information: Engineering compounds for the recovery of critical elements from slags: melt characteristics of Li_5AlO_4, $LiAlO_2$ and $LiAl_5O_8$

Sven Hampel,[†] Iyad Alabd Alhafez,[‡] Thomas Schirmer,[¶] Nina Merkert,[‡] Sophie Wunderlich,[†] Alena Schnickmann,[¶] Haojie Li,[§] Michael Fischlschweiger,[§] and Ursula Elisabeth Adriane Fittschen[*,†]

†*Institute of Inorganic and Analytical Chemistry, Clausthal University of Technology, Arnold-Sommerfeld-Straße 4, 38678 Clausthal-Zellerfeld, Germany*

‡*Institute of Applied Mechanics, Clausthal University of Technology, Arnold-Sommerfeld-Straße 6, 38678 Clausthal-Zellerfeld, Germany*

¶*Department of Mineralogy, Geochemistry, Salt Deposits, Clausthal University of Technology, Adolph-Roemer-Straße 2A, 38678 Clausthal-Zellerfeld, Germany*

§*Technical Thermodynamics and Energy Efficient Material Treatment, Clausthal University of Technology, Agricolastraße 4, 38678 Clausthal-Zellerfeld, Germany*

E-mail: ursula.fittschen@tu-clausthal.de

Phone: +49 (0)5323722205

Figure S1: Rietveld refinement of Li_5AlO_4

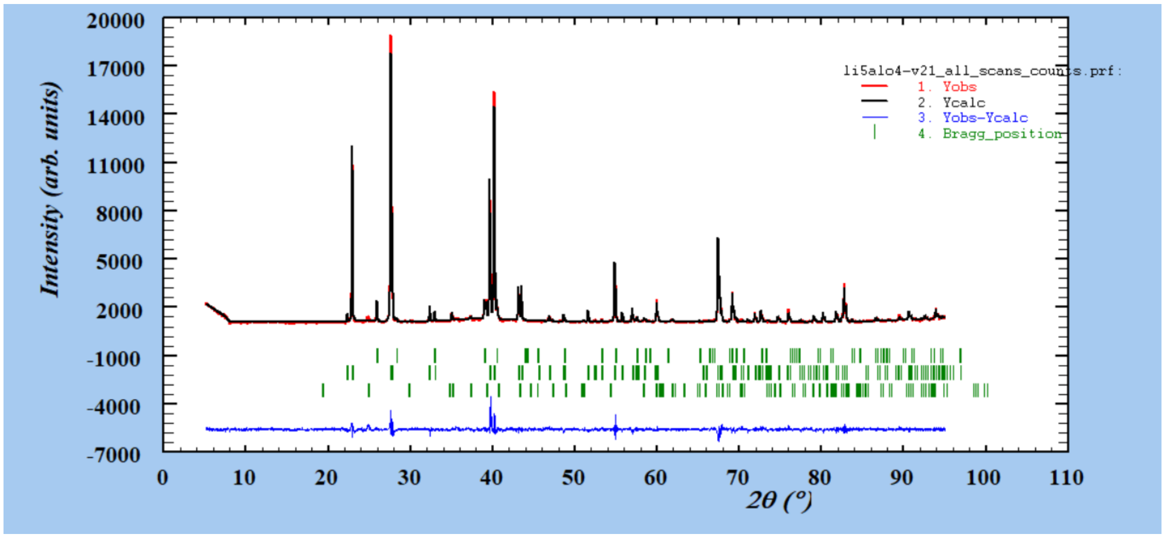

Figure S1: Rietveld refinement (Co Kα = 1.789 Å) of synthesized Li_5AlO_4 with **$LiAlO_2$**, Li_5AlO_4, and $LiOH{\cdot}H_2O$ (from top to bottom)

Figure S2: Mean square displacement of $LiAlO_2$ at 2500 K

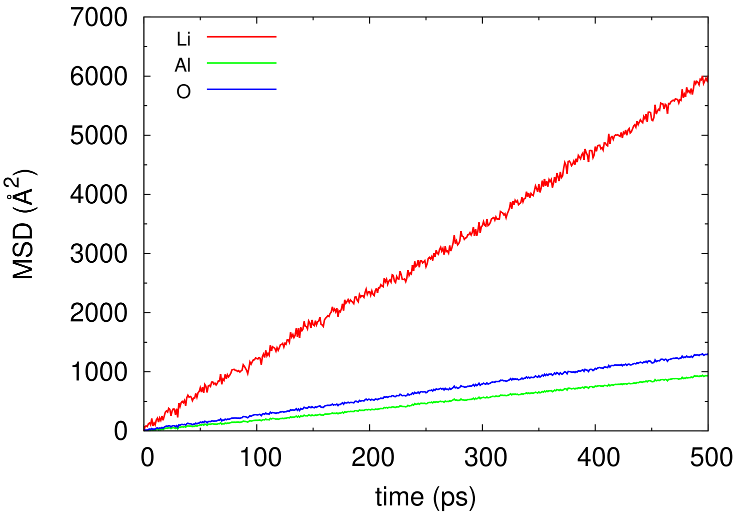

Figure S2: Mean-square displacement of Li^+, Al^{3+} and O^{2-} in $LiAlO_2$ at T = 2500 K

Figure S3: Structural snapshots and RDF of Li_5AlO_4

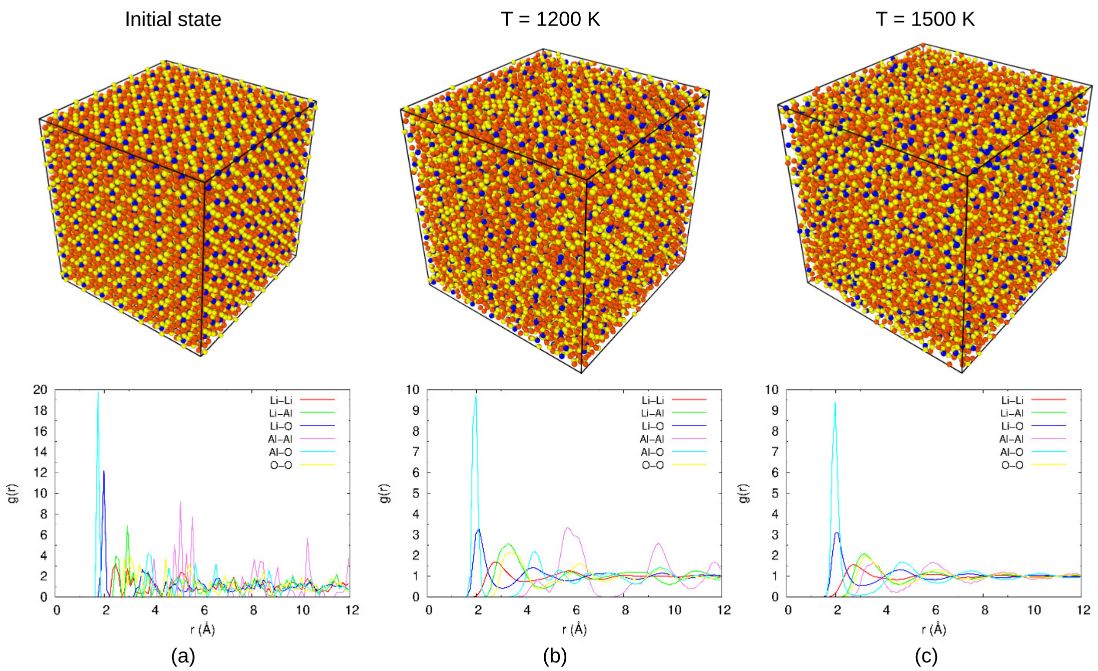

Figure S3: Structural snapshots and radial distribution function (RDF) of Li_5AlO_4 at (a) the initial state at T = 300 K, (b) T = 1200 K, and (c) T = 1500 K

Figure S4: mean square displacement of Li_5AlO_4 at 2500 K

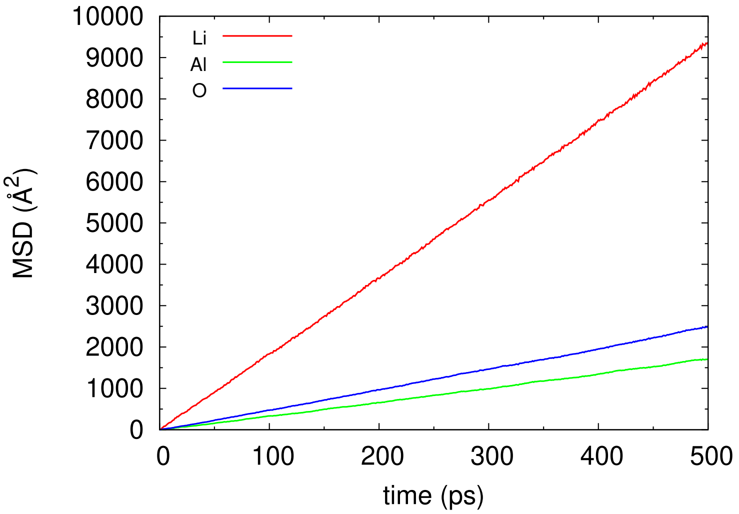

Figure S4: Mean-square displacement of Li^+, Al^{3+} and O^{2-} in Li_5AlO_4 at T = 2500 K

Figure S5: Diffusion coefficient of ion Li_5AlO_4 from 1000 K to 2500 K

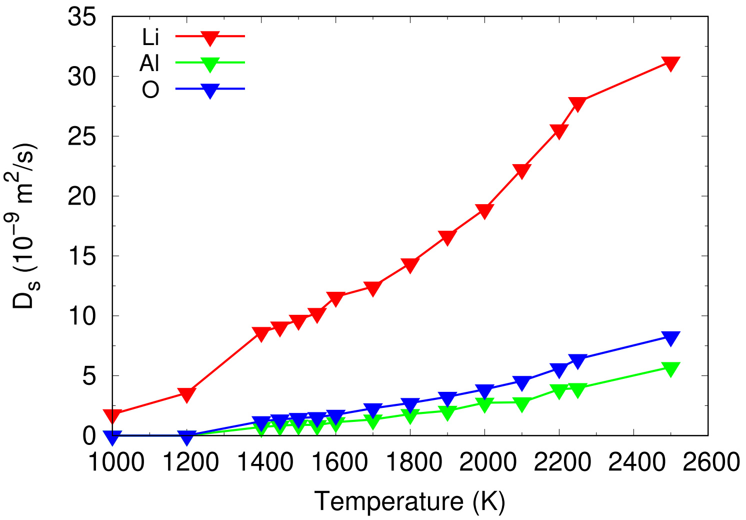

Figure S5: Diffusion coefficient of Li^+, Al^{3+} and O^{2-} in Li_5AlO_4 at temperatures from 1000 K to 2500 K

Figure S6: Structural snapshots and RDF of $LiAl_5O_8$

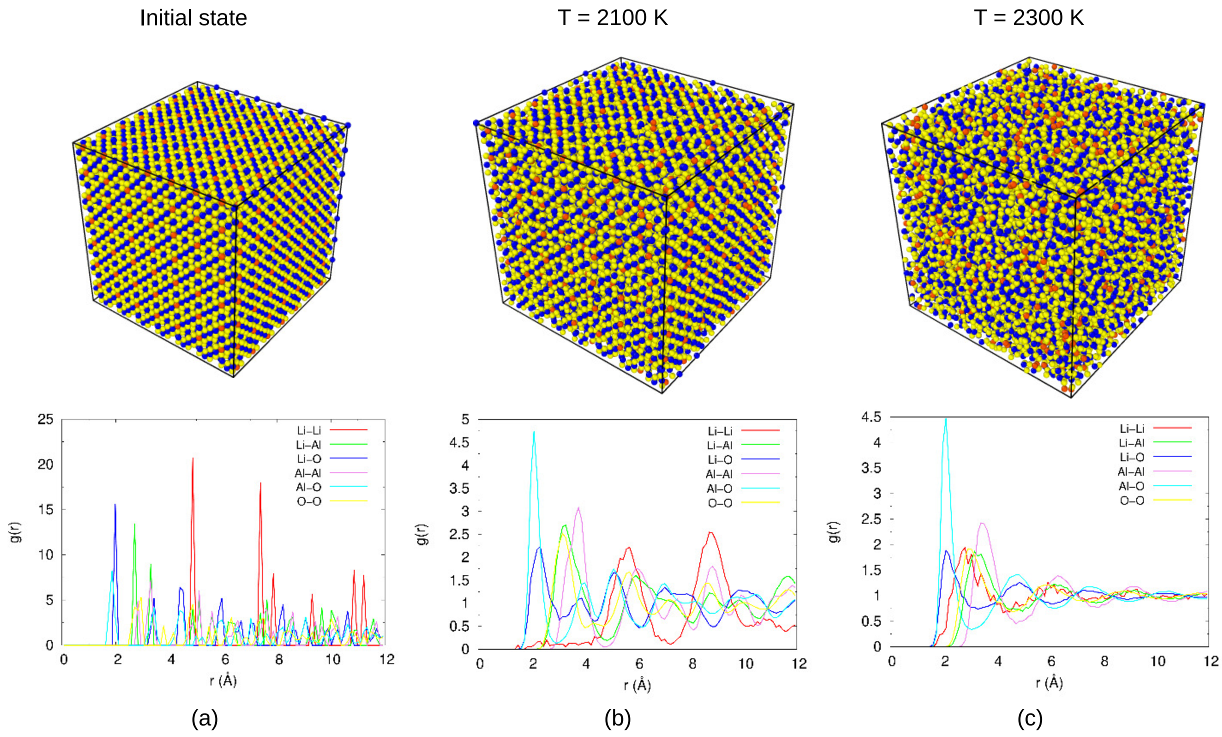

Figure S6: Structural snapshots and radial distribution function (RDF) of $LiAl_5O_8$ at (a) the initial state at T = 300 K, (b) T = 2100 K, and (c) T = 2300 K

Figure S7: Mean square displacement of $LiAl_5O_8$ at 2500 K

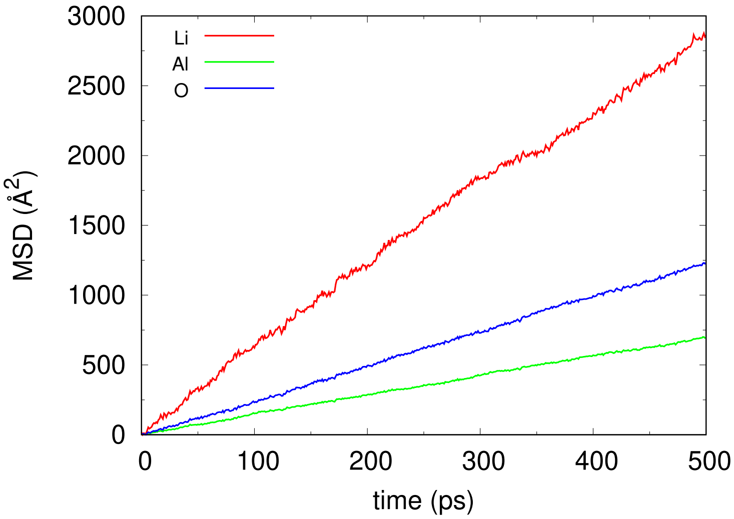

Figure S7: Mean-square displacement of Li^+, Al^{3+} and O^{2-} in $LiAl_5O_8$ at T = 2500 K

Figure S8: Diffusion coefficient of ion $LiAl_5O_8$ from 1500 K to 2750 K

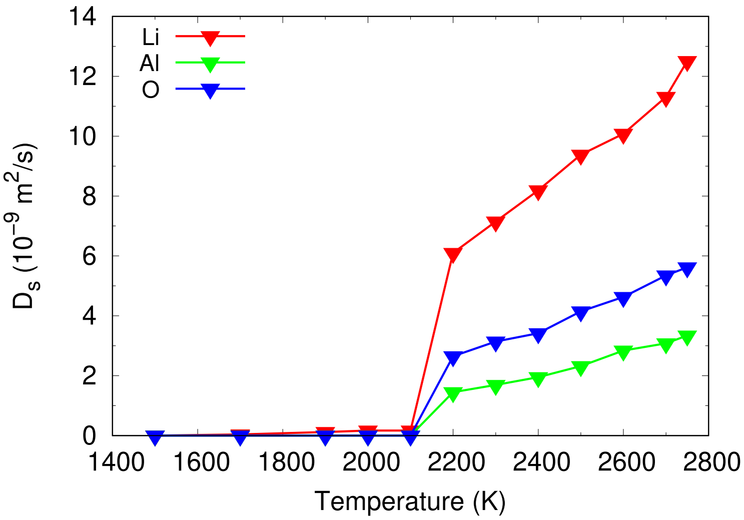

Figure S8: Diffusion coefficient of Li^+, Al^{3+} and O^{2-} in $LiAl_5O_8$ at temperatures from 1500 K to 2750 K
